# Analysis of Hearing Loss and Physical Activity Among US Adults Aged 60-69 Years

**DOI:** 10.1001/jamanetworkopen.2021.5484

**Published:** 2021-04-19

**Authors:** Pei-Lun Kuo, Junrui Di, Luigi Ferrucci, Frank R. Lin

**Affiliations:** 1 National Institute on Aging, National Institutes of Health, Baltimore, Maryland; 2 Department of Biostatistics, Johns Hopkins University Bloomberg School of Public Health, Baltimore, Maryland; 3 Now with Pfizer, Cambridge, Massachusetts; 4 Department of Otolaryngology–Head and Neck Surgery, Johns Hopkins University School of Medicine, Baltimore, Maryland; 5 Cochlear Center for Hearing and Public Health, Johns Hopkins University Bloomberg School of Public Health, Baltimore, Maryland

## Abstract

**Importance:**

Hearing loss may be a modifiable factor associated with decreased physical activity in older adults.

**Objective:**

To examine the association of hearing loss with objectively measured physical activity, including moderate-to-vigorous physical activity, light-intensity physical activity, sedentary behavior, and pattern of physical activity (physical activity fragmentation).

**Design, Setting, and Participants:**

This population-based cross-sectional study used National Health and Nutrition Examination Survey (NHANES) data collected in the 2003 to 2004 cycle and analyzed in 2017 to 2020. Participants aged 60 to 69 years with complete audiometry, physical activity, and comorbidity data were included in the analysis. Data analysis was performed from January 2017 to December 2020.

**Exposures:**

Hearing defined by the pure tone average (PTA; range, 0.5-4 kHz) in the better ear, with normal PTA defined as less than 25 dB hearing loss, mild hearing loss defined as PTA 25 to less than 40 dB hearing loss, and moderate or greater hearing loss defined as a PTA greater than or equal to 40 dB hearing loss.

**Main Outcomes and Measures:**

The primary outcomes were comprehensive metrics of objectively measured physical activity, including time spent in moderate-to-vigorous physical activity, light-intensity physical activity, and sedentary behavior, and physical activity fragmentation. Linear regression was used to model the association between hearing loss and physical activity.

**Results:**

Of the 291 participants (mean [SD] age, 64.53 [2.96] years), 139 (47.8%) were male, 48 (16.5%) had mild hearing loss, and 22 (7.6%) had moderate or greater hearing loss. After adjusting for age, sex, education, race/ethnicity, and comorbidities, hearing loss (vs normal hearing) was significantly associated with less time spent in moderate-to-vigorous physical activity by 5.53 minutes per day (95% CI, −10.15 to −0.90 minutes per day), less time spent in light-intensity physical activity by 28.55 minutes per day (95% CI, −53.07 to −4.02 minutes per day), more time spent in sedentary behaviors by 34.07 minutes per day (95% CI, 8.32 to 59.82 minutes per day), and more fragmented physical activity pattern by 0.38 SD higher in active-to-sedentary transition probability (95% CI, to 0.10 to 0.65). The magnitude of the association of hearing loss (vs normal hearing) with physical activity metrics was equivalent to 7.28 years (95% CI, 3.19 to 11.37 years) of accelerated age for moderate-to-vigorous physical activity, 5.84 years (95% CI, 1.45 to 10.23 years) of accelerated age for light-intensity physical activity, and 10.53 years (95% CI, 2.89 to 18.16 years) of accelerated age for degree of physical activity fragmentation.

**Conclusions and Relevance:**

These findings suggest that hearing loss is associated with a worse physical activity profile. Whether interventions to address hearing loss in adults could improve physical activity profiles will require further study.

## Key Points

**Question:**

Is there an association between hearing loss and physical activity?

**Findings:**

In this cross-sectional study that included 291 adults aged 60 to 69 years, hearing loss was associated with a worse physical activity profile, including less moderate-to-vigorous physical activity, less light-intensity activity, and a more fragmented physical activity pattern.

**Meaning:**

These findings suggest that promoting physical activity among older adults with hearing loss is important, and further research is needed to investigate whether hearing loss interventions could improve physical health profiles.

## Teaser

This cross-sectional study examines the association of hearing loss with objectively measured physical activity, including moderate-to-vigorous physical activity, light-intensity physical activity, sedentary behavior, and physical activity fragmentation.

## Introduction

Physical activity is important to human beings of all ages.^1^ For middle-aged to older adults, physical activity is associated with better quality of life, better physical and cognitive functions, lower risk for cardiovascular diseases, and lower mortality risk.^2,3,4,5,6^ Although there is evidence supporting the health benefits of physical activity, the prevalence of physical inactivity is greater than 15% in the US.^7^ Therefore, it is of vital importance to investigate factors that may contribute to low physical activity.

Hearing impairment may contribute to a lower level of physical activity, either directly, by inability to monitor the environment while being active, or indirectly, by social isolation and increased cognitive load.^8^ A previous study^8^ found that hearing impairment, which is prevalent in nearly two-thirds of adults older than 70 years, is associated with a lower level of physical activity among older adults. Further elucidating the association of hearing impairment with physical activity may offer insights into whether hearing loss is potentially a modifiable factor associated with physical inactivity among older adults.

With recent advances in wearable accelerometers and methods for analyzing these data, physical activity can be measured objectively, thus yielding different parameters of physical activity.^9^ These metrics include conventional summary measures of time spent in various activities (light-intensity physical activity, moderate-to-vigorous physical activity, and sedentary behaviors), as well as 3 more novel metrics, including total activity count for moderate-to-vigorous physical activity and total log-transformed activity count for light-intensity physical activity, which are cutoff-free activity count–based physical activity measures, and patterns of physical activity (ie, activity-to-sedentary transition probability), which reflects the degree of activity fragmentation.^9,10,11^ In this study, we hypothesize that hearing loss, measured by audiometry, is associated with physical activity as quantified by these 3 these novel metrics of objectively measured physical activity among the younger old population (ie, those aged 60-69 years).

## Methods

### Study Design and Participants

The National Health and Nutrition Examination Survey (NHANES) is a nationally representative study in the US of the noninstitutionalized population.^12^ NHANES 1999 to 2004 was approved by the National Center for Health Statistics research ethics review board. All participants provided written informed consent. Since 1999, NHANES has recruited a cross-sectional sample of the US population every 2 years. The analytical cohort for the present cross-sectional study includes 60- to 69-year-old participants from the 2003 to 2004 wave of NHANES when audiometry and actigraphy were both conducted.^12^ Of the 300 participants with valid physical activity measurement and audiometry examinations, 291 participants were included in our analysis after excluding 9 participants without complete comorbidity profile.

The present study was approved by the National Center for Health Statistics. Informed consent was not sought again for this analysis in accordance with 45 CFR §46. This study adheres to the Strengthening the Reporting of Observational Studies in Epidemiology (STROBE) reporting guideline.^13^

### Hearing

In NHANES, trained examiners administered pure tone audiometry tests using established NHANES protocols, based on a modified Hughson-Westlake procedure.^14^ One-half of NHANES participants aged 20 to 69 years were randomly selected to undergo the pure tone audiometry in a sound-isolating room in a mobile examination center, and air conduction hearing thresholds measured in decibels hearing level for each ear across different frequencies were recorded. In our study, hearing function is defined as the 4-frequency pure tone average (PTA) across 0.5, 1, 2, and 4 kHz at the better ear. We defined hearing loss as PTA greater than or equal to 25 dB at the better ear, mild hearing loss as PTA 25 to less than 40 dB in the better ear, and moderate or greater hearing loss as PTA greater than or equal to 40 dB in the better ear.^15^

### Physical Activity

Physical activity was measured objectively using accelerometer-based physical activity monitors. In NHANES, participants were instructed to place a uniaxial accelerometer (ActiGraph AM-7164) on the right hip for 7 consecutive days according to the protocol.^16^ The devices were programmed to record activity counts at 1-minute epochs.^17^ Subjects were included if they had at least 3 valid days, defined as wearing at least 10 hours per day.^18,19,20^ Nonwear time was defined as any interval of 90 minutes or longer of consecutive 0 counts, with an allowance for up to 2 minutes of nonzero counts between 1 and 99.^17,21^ We calculated 3 novel metrics of physical activity: log-transformed total activity counts, total log-transformed activity counts, and physical activity fragmentation.^9,10,11^ Total activity counts and total log-transformed activity counts are cutoff-free physical activity summaries that have been shown to be highly correlated with and share the same distribution with time spent in moderate-to-vigorous physical activity and light-intensity physical activity, respectively, in NHANES.^9^ Log-transformed total activity counts were calculated by taking the logarithm of total activity counts to reduce the skewness of total activity counts.^9,11,22^ Total log-transformed activity counts were calculated by the summation of log-transformed minute-level activity count. The intuition is that log transformation, as a nonlinear transition, shrinks larger minute-level activity counts more than lower minute-level activity counts. Therefore, taking the log before summing up activity counts results in giving relatively more weight to smaller activity count (ie, the physical activity with lower intensity).^9,11^ These 2 summaries have at least 2 strengths: they are cutoff free, making cross-device and cross-cohort comparison possible, and they recognize the heterogeneity within the same category of physical activity. Furthermore, these concepts have been validated in NHANES.^9^ The third novel metric of physical activity is physical activity fragmentation.^10,11^ Physical activity fragmentation is one of the ways to quantify the accumulation pattern of daily physical activity and can be calculated as active-to-sedentary transition probability (with a higher value implying a more fragmented or less sustained physical activity pattern).^10,11^ To provide the most commonly used but cutoff-dependent physical activity summaries, we also calculated the time spent in different categories of physical activity. Time spent in moderate-to-vigorous physical activity, light-intensity physical activity, and sedentary behaviors were calculated as the total time with 2020 or more activity counts per minute, fewer than 2020 and 100 or more activity counts per minute, and fewer than 100 activity counts per minute, respectively.^17,21^

### Other Covariates

Other characteristics of study participants were summarized as collected via interview. Self-reported age was used as a continuous variable, and self-reported sex was used as a binary variable. Levels of education were summarized into 3 groups: less than high school, high school, and higher than high school. Race/ethnicity was classified into 5 categories in NHANES: non-Hispanic White, non-Hispanic Black, Mexican American, other race including multiracial, and other Hispanic. Diabetes, hypertension, overweight, congestive heart failure, coronary heart disease, angina, and heart attack were defined as the participants being told they have the conditions by a doctor or health professional. Chronic obstructive pulmonary disease was defined as answering yes to having chronic bronchitis and/or emphysema. The answers with “don’t know” or “reject” were considered as missing.

### Statistical Analysis

The baseline characteristics were summarized as number (proportion) for the categorical variables and mean (SD) for most continuous variables. Median and interquartile range (IQR) were used for skewed continuous variables. Diurnal changes of physical activity across 3 different groups (no hearing loss, mild hearing loss, and moderate-to-greater hearing loss) were plotted as smoothed curves. To quantify the association of hearing function with physical activity, we used ordinary linear regressions with different detailed physical activity metrics, including log-transformed total activity count, total log-transformed activity count, and active-to-sedentary transition probability as dependent variables and degrees of hearing loss as independent variables. Although log-transformed total activity count and total log-transformed activity count are cutoff-free metrics of physical activity, these physical activity summaries are still new to most clinicians. Therefore, we also used the traditional commonly used physical activity measurements, including time spent in moderate-to-vigorous physical activity, time spent in light-intensity physical activity, and time spent in sedentary behavior, as our dependent variables. To better understand the potential dose-response association between hearing function and physical activity, hearing was used not only as a binary variable indicating whether the participant had hearing loss, but also was treated as the other variable in 2 different ways in the model: first, as a categorical variable representing normal hearing, mild hearing loss, and moderate-to-greater hearing loss, and second, as a continuous variable using PTA. Wearing time for the accelerometer was also adjusted when the outcome might depend on the wearing time. To account for potential confounders, age, sex, levels of education, race/ethnicity, hypertension, diabetes, congestive heart failure, overweight, coronary heart disease, angina, heart attack, and chronic obstructive pulmonary disease were adjusted sequentially. Because the units of novel physical activity metrics were not intuitive, the effect size (standardized coefficient) was reported for these outcomes (log-transformed total activity count, total log-transformed activity count, and active-to-sedentary transition probability). To aid the interpretation of study results, the regression coefficient for hearing with physical activity was scaled by the regression coefficient of chronological age with physical activity and was termed the accelerated age equivalent of hearing on physical activity. Both point estimates and 95% CIs were reported. In the sensitivity analyses, we investigated the other approach to adjust for wearing time by using beta regression to explore the association between hearing and the proportion of time spent in different physical activity categories during the wearing time. The analysis was conducted using R statistical software version 3.6.0 (R Project for Statistical Computing). Data analysis was performed from January 2017 to December 2020.

## Results

Of the 291 participants (mean [SD] age, 64.53 [2.96] years), 139 (47.8%) were male, 48 (16.5%) had mild hearing loss, and 22 (7.6%) had moderate or greater hearing loss (Table 1; eTable 1 and eTable 2 in the Supplement). The median (IQR) time spent in moderate-to-vigorous physical activity was 7.71 (3.08-19.57) minutes per day, that spent in light-intensity physical activity was 326.50 (260.81-382.60) minutes per day, and that spent in sedentary behaviors was 518.43 (438.45-597.86) minutes per day (Table 1).

**Table 1.** Baseline Characteristics of Participants Aged 60 to 69 Years in the National Health and Nutrition Examination Survey, 2003 to 2004

Variable	Participants, No. (%) (N = 291)
Age, mean (SD), y	64.53 (2.96)
Sex	
Male	139 (47.8)
Female	152 (52.2)
Hearing loss category	
None	221 (75.9)
Mild	48 (16.5)
Moderate or greater	22 (7.6)
Pure tone average, decibels	
Median (IQR)	17.50 (12.50-25.00)
Mean (SD)	20.62 (13.11)
Education	
Less than high school	102 (35.1)
High school	61 (21.0)
More than high school	128 (44.0)
Physical activity measurements	
Accelerometer wear time, median (IQR), min/d	852.71 (786.21-917.75)
Time spent in moderate-to-vigorous physical activity, min/d	
Mean (SD)	14.50 (16.77)
Median (IQR)	7.71 (3.08-19.57)
Time spent in light-intensity physical activity, min/d	
Mean (SD)	323.30 (91.30)
Median (IQR)	326.50 (260.81-382.60)
Time spent in sedentary behaviors, min/d	
Mean (SD)	536.80 (162.39)
Median (IQR)	518.43 (438.45-597.86)
Log-transformed total activity count, mean (SD)^a^	12.13 (0.48)
Total log-transformed activity count, mean (SD)^b^	2788.02 (636.82)
Active-to-sedentary transition probability, mean (SD)^c^	0.28 (0.08)

Abbreviation: IQR, interquartile range.

^a^ Log-transformed total activity count is a cutoff-free physical activity measure, calculated by log-transformed total activity count, which is highly correlated with and shares the same distribution of time spent in moderate-to-vigorous physical activity.

^b^ Total log-transformed activity count is another cutoff-free physical activity measure, calculated by the summation of log-transformed minute-level activity count, which is highly correlated with and shares the same distribution of time spent in light-intensity physical activity.

^c^ Active-to-sedentary transition probability is a measurement for pattern of daily physical activity accumulation, with higher values reflecting more fragmented physical activity pattern.

To explore the association between hearing function and physical activity, minute-level diurnal patterns across 3 different groups (no hearing loss, mild hearing loss, and moderate or greater hearing loss) are presented in the Figure. Across the hearing groups, similar patterns of diurnal physical activity were observed over the day (eg, greatest activity in the morning with gradual decreases over the afternoon and evening). However, compared with those with normal hearing, individuals with mild and moderate or greater hearing loss, respectively, had dose-dependent lower activity counts at any time point over the course of the day.

**Figure.**
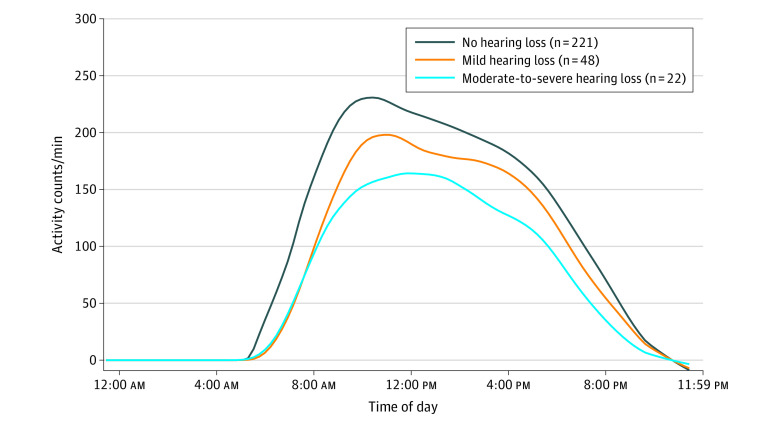
Diurnal Pattern of Physical Activity Stratified by Hearing Function Among 60- to 69-Year-Old Participants in the National Health and Nutrition Examination Survey, 2003 to 2004

In regression analyses (Table 2), after adjusting for age, sex, levels of education, accelerometer wear time, race/ethnicity, hypertension, diabetes, congestive heart failure, overweight, coronary heart disease, angina, heart attack, and chronic obstructive pulmonary disease, every 10 dB increase in hearing loss was associated with less mean time spent in moderate-to-vigorous physical activity by 1.80 minutes per day (95% CI, −3.30 to −0.30 minutes per day), less mean time spent in light-intensity physical activity by 6.68 minutes per day (95% CI, −14.67 to 1.30 minutes per day), more mean time spent in sedentary behaviors by 8.48 minutes per day (95% CI, 0.09 to 16.87 minutes per day), and a more fragmented physical activity pattern by 0.10 SD higher in active-to-sedentary transition probability (95% CI, 0.01 to 0.19). Log-transformed total activity count and total log-transformed activity count are the activity count–based measurement reflecting moderate-to-vigorous physical activity and light-intensity physical activity, respectively. Similar associations between hearing and physical activity were observed for both log-transformed total activity count and total log-transformed activity count (Table 2).

**Table 2.** Association Between Hearing Functions and Objectively Measured Physical Activity Among Participants Aged 60 to 69 Years in the National Health and Nutrition Examination Survey, 2003 to 2004

Outcome	Hearing function measurement, estimate (95% CI)			
	First model	Second model	Third model	
	Continuous measurement, PTA, 10 dB	HL vs no HL	Mild HL vs no HL	Moderate or greater HL vs no HL
Time spent in different categories of physical activity, min/day^a^				
Moderate-to-vigorous physical activity	−1.80 (−3.30 to −0.30)	−5.53 (−10.15 to −0.90)	−5.24 (−10.52 to −0.02)	−6.13 (−13.50 to 1.24)
Light-intensity physical activity	−6.68 (−14.67 to 1.30)	−28.55 (−53.07 to −4.02)	−26.40 (−54.22 to 1.43)	−33.54 (−72.63 to 5.54)
Sedentary behaviors	8.48 (0.09 to 16.87)	34.07 (8.32 to 59.82)	31.67 (2.45 to 60.88)	39.67 (−1.37 to 80.71)
Novel metrics of physical activity^b^				
Log-transformed total activity count^c^	−0.12 (−0.21 to −0.04)	−0.47 (−0.74 to −0.21)	−0.48 (−0.78 to −0.17)	−0.47 (−0.89 to −0.04)
Total log-transformed activity count^d^	−0.09 (−0.17 to −0.004)	−0.34 (−0.60 to −0.08)	−0.31 (−0.61 to −0.02)	−0.42 (−0.83 to −0.003)
Active-to-sedentary transition probability^e^	0.10 (0.01 to 0.19)	0.38 (0.10 to 0.65)	0.37 (0.06 to 0.68)	0.39 (−0.05 to 0.83)

Abbreviations: HL, hearing loss; PTA, pure tone average.

^a^ Model included age, sex, levels of education, wear time, race/ethnicity, hypertension, diabetes, congestive heart failure, overweight, coronary heart disease, angina, heart attack, and chronic obstructive pulmonary disease.

^b^ Outcome variables were scaled to the unit of 1 SD, so the point estimate was interpreted as effect size. Model included age, sex, levels of education, wear time, race/ethnicity, hypertension, diabetes, congestive heart failure, overweight, coronary heart disease, angina, heart attack, and chronic obstructive pulmonary disease for the outcomes log-transformed total activity count and total log-transformed activity count. Model included age, sex, levels of education, race/ethnicity, hypertension, diabetes, congestive heart failure, overweight, coronary heart disease, angina, heart attack, and chronic obstructive pulmonary disease for active-to-sedentary transition probability.

^c^ Log-transformed total activity count is a cutoff-free physical activity measure, calculated by log-transformed total activity count, which is highly correlated with and shares the same distribution of time spent in moderate-to-vigorous physical activity.

^d^ Total log-transformed activity count is another cutoff-free physical activity measure, calculated by the summation of log-transformed minute-level activity count, which is highly correlated with and shares the same distribution of time spent in light-intensity physical activity.

^e^ Active-to-sedentary transition probability is a measurement for pattern of daily physical activity accumulation, with higher values reflecting more fragmented physical activity pattern.

To account for potential nonlinear associations between hearing levels and physical activity, we also conducted analyses with hearing as a binary (no hearing loss vs hearing loss >25 dB) and categorical variable (no hearing loss, mild hearing loss, and moderate or greater hearing loss). Compared with no hearing loss, moderate or greater hearing loss was associated with lower moderate-to-vigorous physical activity measured by log-transformed total activity count (effect size, −0.47; 95% CI, −0.89 to −0.04), light-intensity physical activity measured by total log-transformed activity count (effect size, −0.42; 95% CI, −0.83 to −0.003), and more fragmented physical activity measured by active-to-sedentary transition probability (effect size, 0.39; 95% CI, −0.05 to 0.83). Compared with no hearing loss, mild hearing loss was associated with lower moderate-to-vigorous physical activity measured by log-transformed total activity count (effect size, −0.48; 95% CI, −0.78 to −0.17), lower light-intensity physical activity measured by total log-transformed activity count (effect size, −0.31; 95% CI, −0.61 to −0.02), and more fragmented physical activity measured by active-to-sedentary transition probability (effect size, 0.37; 95% CI, 0.06 to 0.68). After adjusting for age, sex, education, race/ethnicity, and comorbidities, hearing loss (vs normal hearing) was significantly associated with less time spent in moderate-to-vigorous physical activity by 5.53 minutes per day (95% CI, −10.15 to −0.90 minutes per day), less time spent in light-intensity physical activity by 28.55 minutes per day (95% CI, −53.07 to −4.02 minutes per day), more time spent in sedentary behaviors by 34.07 minutes per day (95% CI, 8.32 to 59.82 minutes per day), and more fragmented physical activity pattern by 0.38 SD higher in active-to-sedentary transition probability (95% CI, 0.10 to 0.65) (Table 2).

To better understand the magnitude of the association between hearing and physical activity, the ratio between the coefficients of hearing and age on physical activity was calculated (described as the accelerated age equivalent of hearing function on physical activity) (Table 3). We observed that the magnitude of a 10 dB difference in hearing loss with physical activity was equivalent to 1.79 years (95% CI, 0.30 to 3.29 years) of accelerated age for moderate-to-vigorous physical activity (measured by log-transformed total activity count), 1.56 years (95% CI, −0.30 to 3.43 years) of accelerated age for light-intensity physical activity (measured by total log-transformed activity count), and 2.69 years (95% CI, 5.08 to 0.29 years) of accelerated age for the degree of fragmentation of physical activity pattern (measured by active-to-sedentary transition probability). Having a hearing loss greater than 25 dB (vs those with no hearing loss) was equivalent to 7.28 years (95% CI, 3.19 to 11.37 years) of accelerated age in terms of moderate-to-vigorous physical activity (measured by log-transformed total activity count), 5.84 years (95% CI, 1.45 to 10.23 years) of accelerated age for light-intensity physical activity (measured by total log-transformed activity count), and 10.53 years (95% CI, 2.89 to 18.16 years) of accelerated age for the degree of fragmentation of physical activity (measured by active-to-sedentary transition probability). Additional sensitivity analyses adjusted for accelerometer wearing time by taking the proportion demonstrating consistent results for the association between hearing and time spent in moderate-to-vigorous physical activity, light-intensity physical activity, and sedentary behaviors (eTable 3 in the Supplement).

**Table 3.** Accelerated Age Equivalent of Hearing Status on Physical Activity^a^

Measures of physical activity	Hearing function comparison and accelerated age equivalent (95% CI), y			
	First model	Second model	Third model	
	Per 10 dB increase in PTA	HL vs no HL	Mild HL vs no HL	Moderate to severe HL vs no HL
Time spent in physical activity				
Moderate to vigorous	1.79 (0.30 to 3.29)	5.58 (0.91 to 10.25)	5.34 (0.02 to 10.66)	6.21 (−1.26 to 13.68)
Light intensity	1.56 (−0.30 to 3.43)	6.89 (0.97 to 12.81)	6.42 (−0.35 to 13.19)	8.16 (−1.35 to 17.67)
Time spent in sedentary behaviors	1.61 (0.02 to 3.20)	6.64 (1.62 to 11.65)	6.21 (0.48 to 11.95)	7.78 (−0.27 to 15.84)
Log-transformed total activity count^b^	1.85 (0.55 to 3.15)	7.28 (3.19 to 11.37)	7.31 (2.67 to 11.95)	7.19 (0.67 to 13.70)
Total log-transformed activity count^c^	1.46 (0.06 to 2.86)	5.84 (1.45 to 10.23)	5.36 (0.34 to 10.39)	7.10 (0.05 to 14.16)
Active-to-sedentary transition probability^d^	2.69 (5.08 to 0.29)	10.53 (2.89 to 18.16)	10.39 (1.7 to 19.08)	10.95 (−1.24 to 23.15)

Abbreviations: HL, hearing loss; PTA, pure tone average.

^a^ Accelerated age equivalent was calculated as scaling the coefficient of hearing variable by the coefficient of chronological age. Model included age, sex, levels of education, wear time, race/ethnicity, hypertension, diabetes, congestive heart failure, overweight, coronary heart disease, angina, heart attack, and chronic obstructive pulmonary disease for the outcomes: time spent in moderate-to-vigorous physical activity, time spent in light-intensity physical activity, time spent in sedentary behavior, log-transformed total activity count, and total log-transformed activity count. Model included age, sex, race/ethnicity, hypertension, diabetes, congestive heart failure, overweight, coronary heart disease, angina, heart attack, and chronic obstructive pulmonary disease for active-to-sedentary transition probability.

^b^ Log-transformed total activity count is a cutoff-free physical activity measure, calculated by log-transformed total activity count, which is highly correlated with and shares the same distribution of time spent in moderate-to-vigorous physical activity.

^c^ Total log-transformed activity count is another cutoff-free physical activity measure, calculated by the summation of log-transformed minute-level activity count, which is highly correlated with and shares the same distribution of time spent in light-intensity physical activity.

^d^ Active-to-sedentary transition probability is a measurement for pattern of daily physical activity accumulation, with higher values reflecting more fragmented physical activity pattern.

## Discussion

Our findings demonstrate that, among adults aged 60 to 69 years, hearing loss was independently associated with poorer physical activity, including less moderate-to-vigorous physical activity, less light-intensity physical activity, more sedentary behaviors, and a more fragmented physical activity pattern. The magnitude of these associations appears to be substantial, with mild and moderate-or-greater hearing loss being equivalent to 5.84 and 7.28 years of aging, respectively, in association with light-intensity physical activity. To the best of our knowledge, this is the first study investigating the association between hearing loss and physical activity using comprehensive accelerometer-based physical activity summary measures among a generalizable sample of older adults in the US.

Our findings are generally consistent with those of previous work exploring the association between hearing and physical activity and physical performance. Previous studies^11,23,24^ found that hearing loss is associated with slower gait speed, which may reflect lower physical activity. Curhan et al^25^ demonstrated that self-reported hearing loss was associated with lower questionnaire-based physical activity among women. Gispen et al^8^ found that moderate or greater audiometry-measured hearing impairment was associated with decreased levels of self-reported physical activity among those aged 70 years and older, but mild hearing impairment was not associated with lower levels of self-reported physical activity. Although examining the association between hearing and physical activity using subjective measurements is subject to recall bias, the finding that hearing loss is associated with lower physical activity remains consistent, but the detailed quantification of these associations was not possible in these previous studies.^26,27,28,29,30^

Our analyses incorporating more comprehensive metrics of physical activity may provide additional insights into the association between hearing impairment and physical activity. Gispen et al^8^ also examined the association between audiometry-measured hearing impairment and accelerometer-based physical activity among those aged 70 years and older. They demonstrated that moderate or greater audiometry-measured hearing impairment was associated with decreased levels of accelerometer-based physical activity among those aged 70 years and older, but mild hearing impairment was not associated with lower level of accelerometer-based physical activity.^8^ Although accelerometer-based physical activity was available in the study conducted by Gispen et al,^8^ it was not analyzed in a continuous manner and was used to categorize each study participant into 1 of the 3 groups (inactive, insufficiently active, and sufficiently active). Consequently, the heterogeneity regarding the level of physical activity within each group was masked in previous research.^22^

One of the unique findings from the present study is that hearing loss was associated with more fragmented physical activity, meaning that participants with hearing loss were more likely to break sustained activity into small bouts. Describing the pattern of physical activity using active-to-sedentary transition probability provides a potential way to objectively quantify physical activity by incorporating 3 important components of physical activity: the time spent in active phase, the time spent in sedentary behaviors, and the transition between them. Previous studies^10,11,31^ demonstrated that a more fragmented physical activity pattern, quantified by active-to-sedentary transition probability, is associated with slower gait speed and longer time to finish a 400-m walk independent of the total amount of physical activity, and may be more associated with risk of mortality compared with total amount of physical activity. Our study showed that both mild hearing loss and moderate or greater hearing loss were associated with a more fragmented physical activity pattern among these younger old adults.

Our observations could be explained by several potential mechanisms. First, studies^32,33^ have shown that those with hearing loss may experience greater social isolation, which can further contribute to lower levels of physical activity. Second, hearing loss has been found to be associated with depression, which can also result in lower levels of physical activity.^34^ Third, cognitive load can be higher among those with hearing loss and can result in the inability to maintain in an active state for a long time, leading to a higher probability of transitioning from an active state to a sedentary state and having lower levels of physical activity. Finally, hearing loss has been shown to be associated with a higher risk of falls.^35,36^ For individuals with hearing loss, shortening the consecutive time spent in an active phase with more breaks, which leads to a more fragmented physical activity pattern and lower level of physical activity, could plausibly be a compensatory mechanism to reduce the risk of falling. Our results may also be explained by other possibilities. For instance, some unmeasured factors, such as microvascular disease or some type of neurodegenerative process, could adversely affect hearing, physical function, and physical activity.^37,38^ In our study, our results remained substantively unchanged after adjustment for age, sex, education level, race/ethnicity and comorbidities, but residual confounding remains possible.

### Implications

There are several important implications of our study findings. First, because physical activity provides health benefits, those with hearing loss may experience worse health condition in the future due to lower levels of physical activity. Therefore, promoting healthy aging, including physical activity, among those with hearing loss is of interest to public health. Second, further studies should address whether hearing rehabilitation or the use of hearing aids for those with hearing loss can increase physical activity. Third, a previous study^39^ showed that there are still approximately 20% of primary care practitioners who do not feel prepared to recommend physical activity to patients with disabilities, including hearing limitation. Our results suggest that those with hearing loss may benefit the most from physical activity because they have lower physical activity level and imply that primary care practitioners should promote physical activity more frequently in this population.

### Limitations

There are several limitations of our study. First, we are unable to extensively explore whether the results might be the consequence of common factors, such as neurodegenerative process, because of the lack of neuroimaging information. Second, our study results are based on a modest cohort of 291 participants all aged 60 to 69 years. Whether similar results would be obtained with larger cohorts of adults of more diverse ages remains to be determined. This potential limitation, however, could strengthen the internal validity of our findings given the restricted age range of our study population and the fact that age is likely the most important potential confounder of the association between hearing loss and physical activity. Third, more than 95% of the participants were Hispanic, non-Hispanic White, non-Hispanic Black, and Mexican American. Our results may not be generalizable to other race/ethnicity groups. In addition, those who were unable to remove the hearing aid for testing were excluded per NHANES protocol. Thus, our results might underestimate the association between hearing loss and physical activity.

## Conclusions

In this cross-sectional study, older adults with hearing loss had a poorer physical activity profile, including less moderate-to-vigorous physical activity, less light-intensity physical activity, more sedentary behaviors, and more fragmented physical activity pattern. Further research is needed to explore the underlying mechanism and investigate whether intervention targeting hearing loss could improve physical health profiles.

## Notes

Supplement. **eTable 1.** Physical Activity Baseline of 60-69 Years Old Participants, NHANES 2003-2004 (n = 291) **eTable 2.** Baseline Characteristics of 60-69 Years Old Participants, NHANES 2003-2004 (n = 291), Stratified by Hearing **eTable 3.** Association Between Hearing Functions and Proportion of Time Spent in Different Categories Among 60-69 Year-Old Participants in NHANES 2003-2004
