# Supplementary material for: Analysis of Hearing Loss and Physical Activity Among US Adults Aged 60-69 Years
Source: JAMA Netw Open. 2021 Apr 19;4(4):e215484. doi: 10.1001/jamanetworkopen.2021.5484 (PMC8056278; doi:10.1001/jamanetworkopen.2021.5484)
Supplement: Supplement. — eTable 1. Physical Activity Baseline of 60-69 Years Old Participants, NHANES 2003-2004 (n = 291) eTable 2. Baseline Characteristics of 60-69 Years Old Participants, NHANES 2003-2004 (n = 291), Stratified by Hearing eTable 3. Association Between Hearing Functions and Proportion of Time Spent in Different Categories Among 60-69 Year-Old Participants in NHANES 2003-2004 [file jamanetwopen-e215484-s001.pdf]

Supplemental Online Content

Kuo PL, Di J, Ferrucci L, Lin FR. Analysis of hearing loss and physical activity among US adults aged 60-69 years. *JAMA Netw Open.* 2021;4(4):e215484. doi:10.1001/jamanetworkopen.2021.5484

eTable 1. Physical Activity Baseline of 60-69 Years Old Participants, NHANES 2003-2004 (n = 291)

eTable 2. Baseline Characteristics of 60-69 Years Old Participants, NHANES 2003-2004 (n = 291), Stratified by Hearing

eTable 3. Association Between Hearing Functions and Proportion of Time Spent in Different Categories Among 60-69 Year-Old Participants in NHANES 2003-2004

This supplemental material has been provided by the authors to give readers additional information about their work.

© 2021 Kuo PL et al. *JAMA Network Open.*

eTable 1. Physical Activity Baseline of 60-69 Years Old Participants, NHANES 2003-2004 (n = 291)

Variable	Median (IQR)
Physical Activity Measurements	
Average wear time (minutes/day)	852.71 [786.21, 917.75]
Proportion of time spent in moderate-to-vigorous physical activity (%)	0.9 [0.4, 2.2]
Proportion of time spent in light-intensity physical activity (%)	36.9 [30.6, 44.4]
Proportion of time spent in sedentary behavior (%)	61.1 [53.4, 67.8]

Abbreviations: IQR = interquartile range

© 2021 Kuo PL et al. *JAMA Network Open.*

eTable 2. Baseline Characteristics of 60-69 Years Old Participants, NHANES 2003-2004 (n = 291), Stratified by Hearing			
	No HL (N= 221)	Mild HL (N = 48)	Moderate to severe HL (N = 22)
	n (%) / mean (SD) / median (IQR)		
Age (years), mean (SD)	64.29 (2.96)	64.82 (2.94)	66.26 (2.51)
Male, n (%)	88 (39.8)	33 (68.8)	18 (81.8)
PTA (median [IQR]), dB	15.00 [11.25, 18.75]	28.75 [27.50, 32.50]	45.62 [41.25, 62.50]
Education, n (%)			
Less than High School	68 (30.8)	24 (50.0)	10 (45.5)
High School	41 (18.6)	14 (29.2)	6 (27.3)
More than High School	112 (50.7)	10 (20.8)	6 (27.3)
Physical Activity Measurements			
Time spent in moderate-to-vigorous physical activity (minutes/day)	8.14 [3.71, 20.43]	6.88 [2.81, 16.44]	3.57 [2.61, 9.39]
Time spent in light intensity physical activity (minutes/day)	329.83 [270.29, 385.67]	322.50 [241.72, 362.70]	247.57 [202.61, 325.41]
Time spent in sedentary behaviors (minutes/day)	503.57 [425.29, 580.86]	527.14 [457.69, 617.54]	573.00 [477.54, 650.00]
Total log-transformed activity count	2859.25 (617.77)	2655.18 (626.73)	2362.32 (662.90)
Log-transformed total activity count	12.19 (0.45)	11.98 (0.52)	11.88 (0.54)
Active-to-sedentary transition probability	0.27 (0.07)	0.30 (0.08)	0.31 (0.07)
Average wear time (minutes/day)	853.29 [789.50, 916.00]	854.67 [783.07, 940.50]	808.76 [785.04, 930.15]
Proportion of time spent in moderate-to-vigorous physical activity (%)	0.97 [0.43, 2.41]	0.86 [0.33, 1.73]	0.45 [0.24, 1.35]
Proportion of time spent in light-intensity physical activity (%)	37.92 [31.83, 45.78]	36.14 [27.53, 40.71]	29.98 [23.69, 37.94]
Proportion of time spent in sedentary behavior (%)	59.98 [52.52, 66.78]	62.56 [58.29, 72.17]	68.36 [61.42, 75.93]
Comorbidity, n (%)			
Hypertension	119 (53.8)	29 (60.4)	13 (59.1)
Diabetes	36 (16.3)	9 (18.8)	8 (36.4)
Congestive heart failure	8 (3.6)	5 (10.4)	4 (18.2)
Overweight	95 (43.0)	18 (37.5)	6 (27.3)
Heart attack	11 (5.0)	9 (18.8)	4 (18.2)
Chronic obstructive pulmonary disease	14 (6.3)	3 (6.2)	2 (9.1)

© 2021 Kuo PL et al. *JAMA Network Open.*

Angina	19 (8.6)	3 (6.2)	6 (27.3)
Race/Ethnicity, n (%)			
Non-Hispanic White	113 (51.1)	23 (47.9)	13 (59.1)
Non-Hispanic Black	39 (17.6)	10 (20.8)	1 (4.5)
Mexican American	58 (26.2)	11 (22.9)	6 (27.3)
Other Race - Including Multi-Racial	8 (3.6)	3 (6.2)	1 (4.5)
Other Hispanic	3 (1.4)	1 (2.1)	1 (4.5)

© 2021 Kuo PL et al. *JAMA Network Open.*

eTable 3. Association Between Hearing Functions and Proportion of Time Spent in Different Categories Among 60-69 Year-Old Participants in NHANES 2003-2004

Outcome: Proportion of time spent in different categories of physical activity [a]			
Physical Activity Category	Moderate-to-vigorous physical activity	Light intensity physical activity	Sedentary behaviors
Hearing Function Measurement			
	Estimate (95% CI)	Estimate (95% CI)	Estimate (95% CI)
**Continuous Measurement**			
PTA (10dB)	-0.11 (-0.19, -0.04)	-0.03 (-0.08, 0.01)	0.04 (0.001, 0.09)
**Binary HL**			
No HL	Ref	Ref	Ref
HL	-0.34 (-0.58, -0.11)	-0.14 (-0.27, -0.02)	0.17 (0.04, 0.30)
**Category HL**			
No HL	Ref	Ref	Ref
Mild HL	-0.31 (-0.58, -0.05)	-0.14 (-0.28, 0.00)	0.17 (0.02, 0.31)
Moderate or greater HL	-0.41 (-0.79, -0.03)	-0.15 (-0.35, 0.05)	0.18 (-0.03, 0.39)

Abbreviations: HL = hearing loss; PTA = pure tone average

[a] Beta regression was used for this sensitivity analysis because the main dependent variable is proportion (the proportion of time spent in the specific physical activity category among the total time wearing physical activity monitor). The estimate refers to the point estimate in beta regression. The Model included age, sex, levels of education, race/ethnicity, hypertension, diabetes, congestive heart failure, overweight, coronary heart disease, angina, heart attack, and chronic obstructive pulmonary disease.

© 2021 Kuo PL et al. *JAMA Network Open.*
